# Viral Diseases Explorer: a webtool to identify viral disease information derived from multiple LLMs

**DOI:** 10.1093/bioinformatics/btaf613

**Published:** 2025-11-08

**Authors:** Oscar Rojas Labra, Carla M Martinez-Garcia, Nelly Santoyo-Rivera, Daniel Montiel-Garcia, Vijay S Reddy

**Affiliations:** The Hormel Institute, University of Minnesota, Austin, MN 55912, United States; Departments of Computer Systems and Information Technologies, Tecnologico Nacional de Mexico & Instituto Tecnológico Superior de Irapuato, Irapuato, es 36821, Guanajuato, Mexico; Departments of Computer Systems and Information Technologies, Tecnologico Nacional de Mexico & Instituto Tecnológico Superior de Irapuato, Irapuato, es 36821, Guanajuato, Mexico; Department of Integrative Structural and Computational Biology, The Scripps Research Institute, La Jolla, CA 92037, United States; The Hormel Institute, University of Minnesota, Austin, MN 55912, United States; Department of Integrative Structural and Computational Biology, The Scripps Research Institute, La Jolla, CA 92037, United States

## Abstract

**Motivation:**

With nearly 266,500 viruses cataloged currently at NCBI and increasing by the day, identifying the diseases they cause, and the affected hosts remains challenging. Hence the motivation for undertaking this study.

**Results:**

Utilizing the AI-powered tools, we obtained disease information for 165 363 viruses, identifying 2833 unique diseases affecting 5503 distinct hosts and designated consensus diseases confirmed by three LLMs. Using this information, we developed the Viral Diseases Explorer webtool that enables the searches of diseases linked to specific viruses, viruses causing particular diseases, and viruses infecting specific hosts.

**Availability and implementation:**

Viral Diseases Explorer tool can be accessed from the URL: https://virus-world.org/viral_diseases_explorer.php

## 1 Introduction

**Figure 1.**
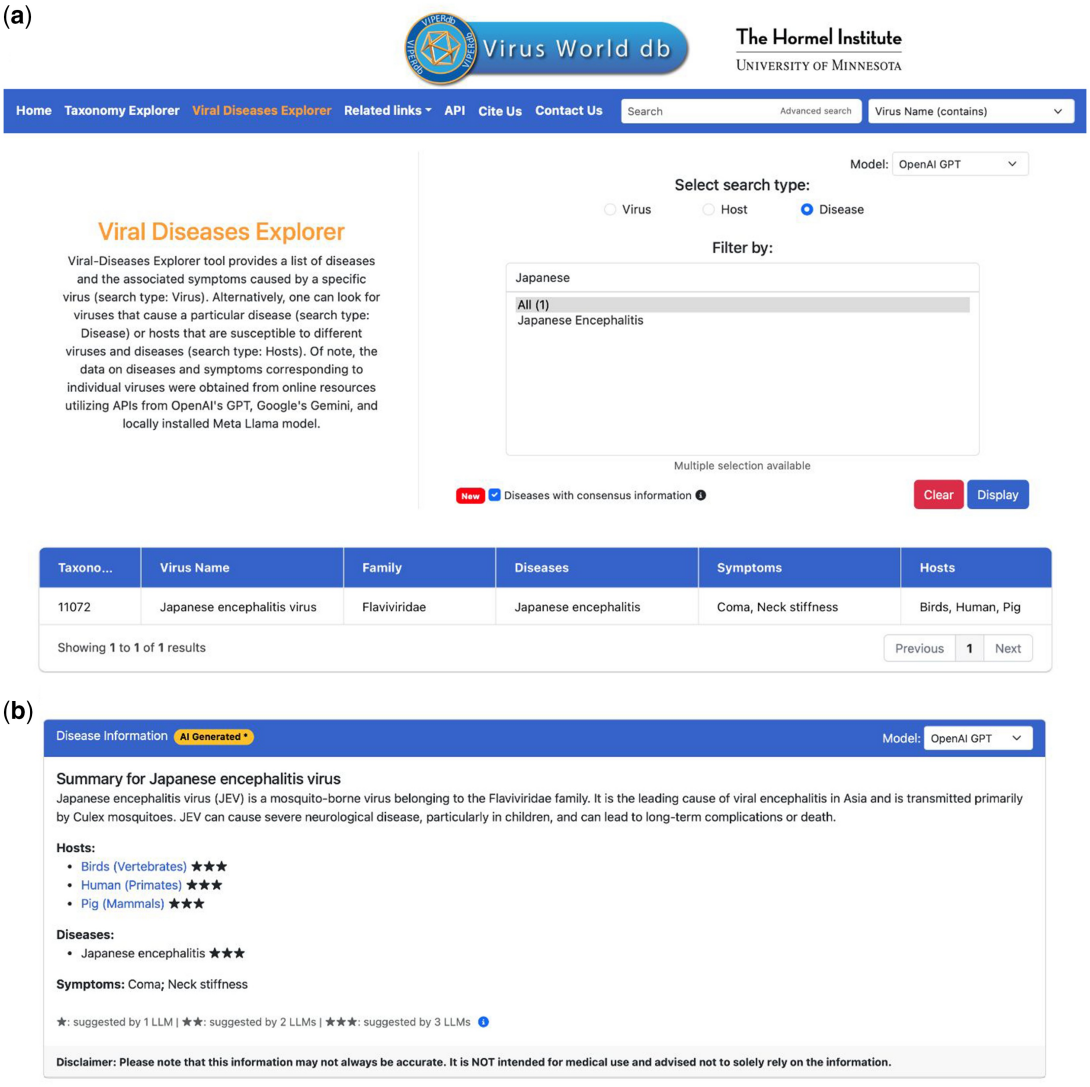
Viral Disease Explorer (VDE) interface. (a) Web-based graphical user interface (GUI) of VDE that allows the searches based on the names or keywords of virus, host or diseases and also permits the choice of different LLMs: OpenAI GPT, Google Gemini or Meta’s Llama. Additionally, a checkbox is provided to select consensus diseases and hosts identified by the three LLMs. The results of search of diseases with a keyword: Japanese identified by the three LLMs are shown. (b) The disease Information of Japanese encephalitis virus that includes a brief summary about the virus, associated diseases, affected hosts and symptoms. Furthermore, the hosts and diseases are listed separately and identified by the star-rating as a confidence (reliability) metric.

Currently, the number of identified viruses (∼266 500) documented at NCBI is increasing on a regular basis. However, the way they are catalogued at NCBI, determining the number of virus families, genera and their constituent viruses is challenging (Sayers *et al.* 2021). Recognizing this unmet need, we created a web resource, Virus World database (VWdb) (https://virus-world.org) (Rojas Labra *et al.* 2023) that contains the curated data on virus taxonomy lineage, genome type and accession codes for the sequence, structural and taxonomy information obtained from various public databases: NCBI (Sayers *et al.* 2021), ICTV (Walker *et al.* 2022), UniProtKB (The UniProt Consortium 2023), GenBank (Sayers *et al.* 2020), ViralZone (Hulo *et al.* 2011), PDB (Berman *et al.* 2000), EMDB (The wwPDB Consortium 2024), and VIPERdb (Carrillo-Tripp *et al.* 2009, Montiel-Garcia *et al.* 2021). Furthermore, we developed a webtool namely Taxonomy Explorer (https://virus-world.org/taxonomy_explorer.php) that makes it easy to select viruses in a chosen family, genus or containing a particular genome type based on the ICTV/NCBI genome classification as well as the Baltimore convention (e.g. ssRNA, dsDNA, etc.). Additionally, one can search and download viruses and the associated metadata in Excel, CSV or JSON formats. Of note, behind the scenes database (VWdb) of the Taxonomy Explorer is updated weekly based on the taxonomy data downloaded directly from NCBI and obtaining related information from the above-mentioned databases. In addition, an information page known as pathogen data sheet (PDS) of each virus is generated dynamically displaying the various information related to virus taxonomy, sequence and structural data (Fig. 1, available as supplementary data at *Bioinformatics* online).

However, we quickly realized the need for missing disease-related information of each virus and the susceptible hosts in the PDS. Recognizing such information cannot be gathered from a single source, we decided to obtain the disease-related information using various Artificial Intelligence (AI) based Large Language Models (LLMs) that are trained on a large amount of data available online. It is notable that the recent rise of AI powered tools has greatly enhanced our ability to rapidly search and obtain the information of interest in a predefined format (e.g. JSON). By querying multiple LLMs, we obtained at least one disease connected to 7505 viruses classified at the taxonomy rank of species.

Here we describe the workflow and “prompt engineering” procedures used to search and acquire the disease information using the Application Programming Interface (API) tools from OpenAI, Google and Meta AI and store the data into separate databases with a specific schema for each LLM and further consolidate the data by postprocessing (Fig. 2, available as supplementary data at *Bioinformatics* online).

## 2 Methods

### 2.1 Acquisition of disease-related data

We used APIs from OpenAI (https://platform.openai.com), Google (https://ai.google.dev) and Meta AI (https://www.llama.com) to search and acquire the disease-related information for viruses at the lineage rank of species. In addition to a brief summary of the virus lineage, we obtained a list of different diseases that a particular virus causes, associated symptoms, the affected hosts and a few related references separately in JSON format. To maintain data integrity and for convenience, the information from OpenAI, Google, and Meta AI were stored in three separate databases. Figure 1 shows the workflow for gathering disease-related information. This involves three main steps—(i) data preparation, (ii) model execution, and (iii) postprocessing of the results. Of note, the data acquisition using the APIs from OpenAI required establishing a pay-as-you-go account, while the use of APIs from Google and Meta were free of charge. Particularly, the queries using Meta’s Llama were done on a local server. Each of the three steps are described in detail below.

### 2.2 Data preparation

This step involves selecting viruses and their taxonomy identifiers (NCBI:txids) at the rank of species from VWdb for which the disease information to be obtained along with the unique virus entries from VIPERdb (https://viperdb.org). VIPERdb is a virus structural database, that contains viruses some of which are classified at the lineage level of sub-species (Carrillo-Tripp *et al.* 2009, Montiel-Garcia *et al.* 2021). Together, a total of 43 177 viruses were selected that do not include bacteriophages (except for those in VIPERdb) and clustered into groups based on the similarity of their names. Before clustering, the virus names were preprocessed by (i) removing any characters/numbers after the word/string “virus” in the name (e.g. 87 was removed in Human rhinovirus 87), (ii) removing extraneous characters (e.g. “(,” “[,” “:,” “;”), (iii) converting the text into lowercase, and (iv) removing the standalone word “virus” from the virus name (e.g. only “Chlorella” was considered in “Chlorella virus”). Of note, these preprocessed names are always linked to their original names stored in a database. The preprocessed names are then encoded into vectors using a sentence transformer model, all-MiniLM-L6-v2 and followed by clustering them using DBSCAN (Density-Based Spatial Clustering of Applications with Noise) (Ester *et al.* 1996). This resulted in 19 482 clusters yielding 19 793 representative viruses, selecting one virus per cluster or one per family when clusters contained members from multiple families. Of which, 3740 clusters contained more than one virus, the rest (majority) consist of a single virus. For example, “Chlorella virus,” “Chlorella virus BW1,” and “Chlorella virus XW01” are grouped into a single cluster named under “Chlorella virus.” Subsequently, batch requests (JSON objects) were assembled to query the LLMs from OpenAI for the disease information (see below) (Fig. 2, available as supplementary data at *Bioinformatics* online). Whereas the information from Google and Meta AI was obtained using the real-time API calls that contain similar prompts as in the above JSON object, to online and local servers, respectively.

### 2.3 Model execution

This step applies only for OpenAI that entails uploading batch request files into the cloud storage at OpenAI and receiving the corresponding file identifiers. This is followed by executing the batch API requests by specifying the file identifiers (*client.batches.create(input_file_id=batch_input_file_id, endpoint=“/v1/chat/completions”, completion_window=“24h”*) (Fig. 3, available as supplementary data at *Bioinformatics* online). The status of completion of the batch jobs was frequently checked (*client.batches.retrieve(“batch_abc123”).status*) until the results were obtained.

*Postprocessing:* The output JSON objects of the batch jobs were downloaded onto a local computer from the cloud storage using an API (*file_response = client.files.content(“file-xyz123”)*). An example output response is shown in Fig. 4, available as supplementary data at *Bioinformatics* online. The output responses from all three LLMs were further processed using various Python scripts and stored into corresponding MySQL databases for ensuing data curation and analysis (Fig. 2, available as supplementary data at *Bioinformatics* online).

### 2.4 Acquisition, curation, and analysis of the susceptible hosts and their NCBI:txids

We initially classified the potential host information obtained from the three LLMs into nine divisions of organisms (bacteria, invertebrates, mammals, phages, plants and fungi, primates, rodents, viruses and vertebrates) as categorized by NCBI. This step involved acquiring the scientific name for each host using primarily Google’s Gemini as it performed better than the others. The scientific names were then used to classify the host organisms into above nine divisions using Gemini (Fig. 5, available as supplementary data at *Bioinformatics* online). After that, both the scientific names and “raw” hostnames obtained from LLMs were preprocessed by removing the punctuation marks and converting the names into lowercase as previously described for clustering of viruses. The flowchart of the host data processing and analysis are illustrated in Fig. 6, available as supplementary data at *Bioinformatics* online. The preprocessed names are then encoded into vectors using a sentence transformer model as previously described. These embeddings were matched with the locally created vector database containing all the organisms from nine divisions composed of 2 545 881 unique entries (NCBI:txids) from NCBI. In addition, we also considered alternate names for each organism that include, scientific name, common name, equivalent name, etc. Initially, we looked for exact matches in the NCBI organism vector database in all divisions for either raw or scientific hostnames obtained from different LLMs. The exact matches include 6582, 4130, and 3462 from OpenAI, Google and MetaAI, respectively. Of note, the search for exact matches in all divisions was done to reduce the chances of any misclassification into a wrong division by Google. The hosts for which we did not find exact matches were searched again for the closest match in the corresponding division of organisms irrespective of their taxonomy rank. For example, the hostname “setosphaeria turcica” obtained from a LLM was matched with “setosphaeria turcica et28a” in the division of Plants and Fungi in the NCBI vector database. Significantly, this matching of the hostnames allowed us to assign a NCBI: txid and obtain the associated lineage information for each hostname from the LLMs. Once NCBI:txids are assigned to the respective hosts, the lower ranked organisms (e.g. subspecies, strain, etc.) were coalesced to the level of species rank in their taxonomy tree. For example, common name “dogs” having the scientific name “Canis Lupus Familiaris” identified as a subspecies is merged to “Canis Lupus” at the species level. Thus, knowing the NCBI: txid allowed us to seamlessly identify the parents and descendants of an organism at any lineage level. It is noteworthy that this was made possible by the lineage organization encoded into the host/organism taxonomy tree created locally from the entire taxonomy data dump available at NCBI (https://ftp.ncbi.nlm.nih.gov/pub/taxonomy/new_taxdump/). By doing this, each host in the VDE database is assigned a NCBI: txid that in turn allowed us to identify all the diseases affecting a particular host lineage.

### 2.5 Clustering and identification of consensus diseases identified by three LLMs

Initially, all the disease names obtained from three LLMs were combined irrespective of their etiological agents (viruses), 5768 in total, and simplified them by removing the annotations indicated in the parenthesis or any host specific information [e.g. “Gastrointestinal infections (specific symptoms not well-documented)” is simplified to “Gastrointestinal infections”]. This simplification was done using a smaller LLM, Qwen 2.5 (32b) from Alibaba that was installed on a local computer. The simplified disease names were then embedded using a sentence transformer, PubMedBERT (Gu *et al.* 2020) and clustered using DBSCAN. This process resulted in 2833 clusters that contained similar diseases ranging from 1 to 486 (e.g. respiratory infections and respiratory disease are clustered together). Each cluster was then identified by a common (consensus) name based on the diseases that constitute a cluster. For example, the above cluster was given a common name, “respiratory diseases.” These consensus disease names, and the associated cluster-ids (integers) were linked back to the viruses that are responsible for causing these diseases. This is followed by further analyzing the overlap of viral diseases provided by the three LLMs for a given virus.

Using the above consensus disease names and associated cluster-ids, and the LLMs from which the information was obtained, we identified various connections between the viruses and the diseases. These data were in turn used to generate Venn diagrams to classify the virus-disease connections that are common between three or two LLMs or found only in one of the LLMs. We found 818 distinct virus-disease connections that are common to three LLMs (Fig. 7, available as supplementary data at *Bioinformatics* online). Furthermore, by linking the host information associated with the virus, we identified 538 virus-disease connections that have a common host also identified by the three LLMs [tripartite (virus–disease–host) connections, Fig. 7, available as supplementary data at *Bioinformatics* online, Table 1, available as supplementary data at *Bioinformatics* online]. Based on this we identified 108 unique hosts. Out of which, 55 virus-disease connections belong to humans containing 54 unique viruses causing 36 distinct diseases (Table 2, available as supplementary data at *Bioinformatics* online).

## 3 Results and discussion

Using these procedures (see Section 2), we obtained the disease information for a combined 165 363 viruses that include 7505 viruses at the rank of species and their descendants. In addition to a brief summary about the virus, the associated diseases, resulting symptoms and the affected hosts along with a few references were obtained. Using these data, we created a webtool termed Viral Diseases Explorer (VDE) (https://virus-world.org/viral_diseases_explorer.php) that allows searches based on the diseases caused by a chosen virus; viruses that cause a particular disease; or all the viruses and associated diseases that affect a particular host (Fig. 1). By clicking on a virus or disease of interest in the list creates a pathogen disease sheet (PDS) for virus. Moreover, using VDE, users can select different LLMs from which the disease information was collected. Significantly, we also included “star-rating” as a confidence metric for the diseases and hosts identified by the number of LLMs in the PDS pages. For example, three stars are given for the diseases or hosts identified by all three LLMs, two stars for the consensus information from two out three LLMs, etc. Of note, because of the consistent errors found in the references (citations) provided by the LLMs, we decided not to include them as part of the PDS pages.

Furthermore, we assigned distinct taxonomy identifiers (NCBI:txids) for various affected hosts as categorized by NCBI using a vector database. This allows the identification of viruses and diseases affecting a particular host lineage. We identified 2833 unique diseases, affecting distinct 5503 hosts, indicating multiple hosts are affected by the same (similar) disease. For example, humans and cattle are affected by Cowpox disease (Table 1, available as supplementary data at *Bioinformatics* online). We identified 329 diseases common to all the three LLMs, while additional 500 diseases are identified by at least two of the three LLMs. Similarly, three LLMs identify 1807 overlapping hosts, while additional 1596 are common to at least in two LLMs. We further analyzed these data to identify the consensus virus–disease–host (tripartite) connections, establishing 538 such connections are common to all three LLMs (Table 1, available as supplementary data at *Bioinformatics* online, Fig. 7, available as supplementary data at *Bioinformatics* online). Additionally, we found that 1532 tripartite connections are identified by at least two of the three LLMs. Using the tripartite connections, we found 54 (unique) human viruses causing distinct 36 diseases (Table 2, available as supplementary data at *Bioinformatics* online). We now provide an option to select consensus virus–disease–host information from multiple LLMs in VDE.

Thus, this study provides a framework to obtain useful information on pathogens, diseases they cause and affected hosts using commonly available AI powered tools and validating them by analyzing consensus results from multiple LLMs. Significantly, these methodologies can be used to update the disease information on regular intervals (e.g. every 3 months), by leveraging the newly released LLMs. Going forward, we plan to apply similar procedures to obtain disease information on other pathogens such as bacteria and archaea. We believe that such information will be useful in the fight against the pathogens and the potential diseases they cause.

## Supplementary Material

btaf613_Supplementary_Data

## Data Availability

The data underlying this article are available and downloaded from the website (https://virus-world.org/viral_diseases_explorer.php).
