# Supplementary material for: Viral Diseases Explorer: a webtool to identify viral disease information derived from multiple LLMs
Source: Bioinformatics. 2025 Nov 8;41(11):btaf613. doi: 10.1093/bioinformatics/btaf613 (PMC12627403; doi:10.1093/bioinformatics/btaf613)
Supplement: btaf613_Supplementary_Data [file btaf613_supplementary_data.zip › Supplementary Information_V4.pdf]

Supplementary Information for

Viral Diseases Explorer: A webtool to identify viral disease information derived from multiple LLMs

Oscar Rojas Labra[1], Carla M. Martinez-Garcia[2], Nelly Santoyo-Rivera[2], Daniel Montiel-Garcia[3] and Vijay S. Reddy[1,3*]

[1]The Hormel Institute, University of Minnesota, Austin, MN 55912, USA

[2]Departments of Computer Systems and Information Technologies, Tecnologico Nacional de Mexico & Instituto Tecnológico Superior de Irapuato, Irapuato, Guanajuato, Mexico.

[3]Department of Integrative Structural and Computational Biology, The Scripps Research Institute, La Jolla, CA 92037, USA

[*] **Correspondence to:**
Vijay S. Reddy, PhD.
The Hormel Institute, University of Minnesota
801 16th Avenue NE
Austin, MN 55912, USA.
E-mail: vsreddy@umn.edu
Tel: +1 (507) 437-9647

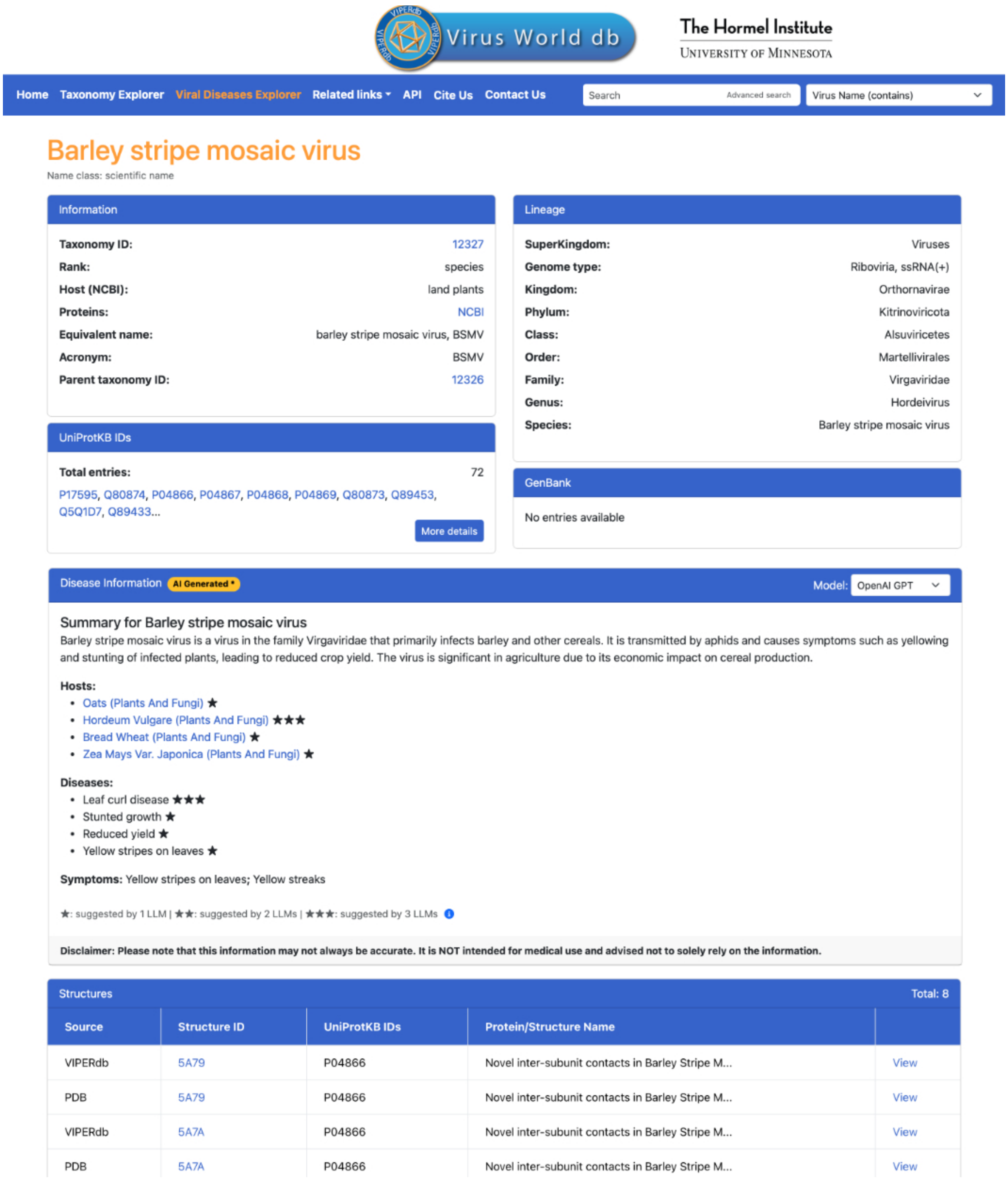

Supplementary Figure 1. An example pathogen data sheet (PDS). A PDS for Barley stripe mosaic virus showing the details of NCBI:TxID with links to NCBI, acronyms, equivalent names, affected hosts (top-left). The taxonomy lineage information is shown on top-right. Shown in the middle is the disease information obtained from the AI agents, identified with the star-rating and shown at the bottom is a table of 3D structures related to BSMV available from various databases (e.g., PDB, VIPERdb etc.)

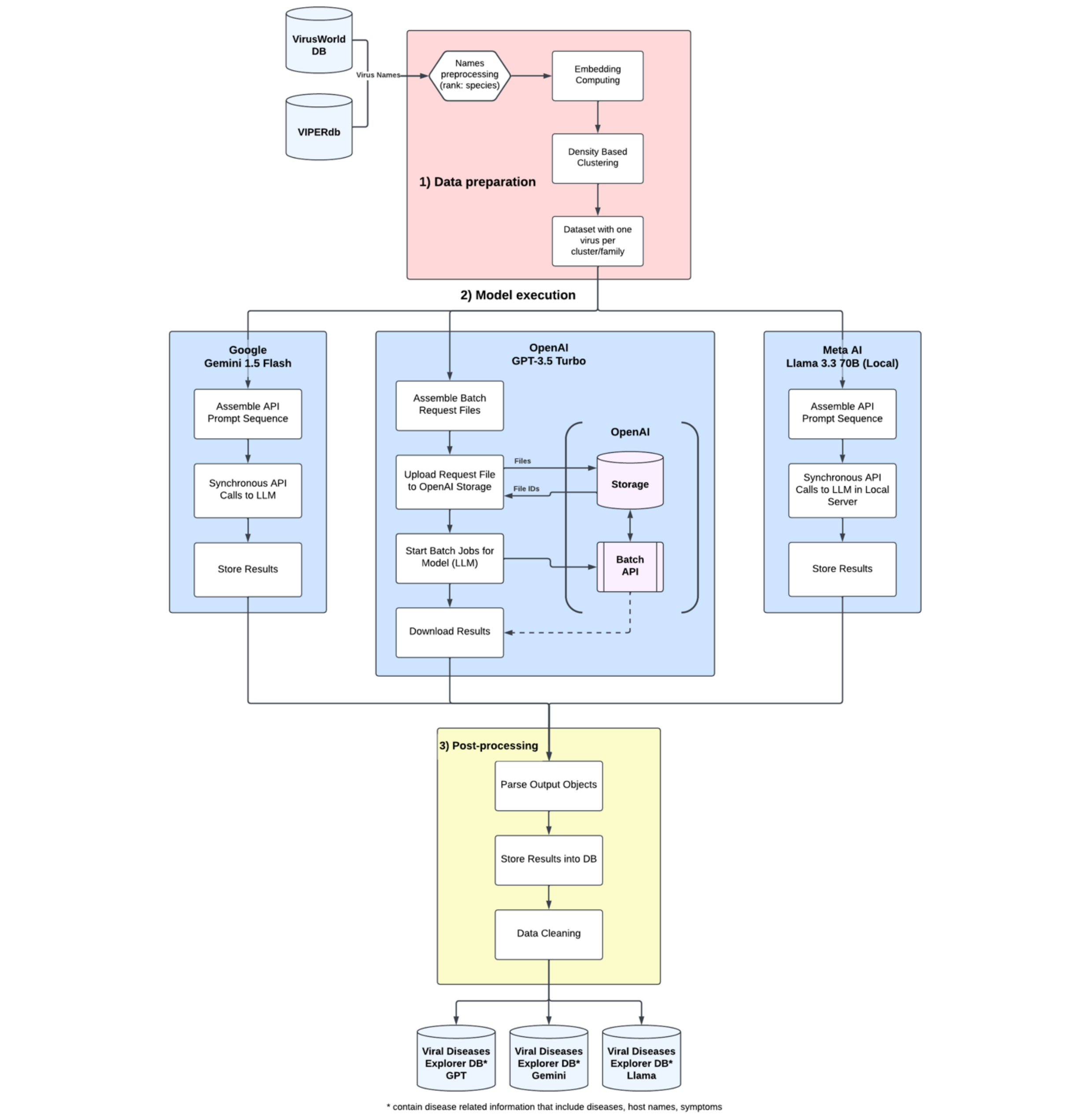

Supplementary Figure 2. Workflow of disease acquisition from LLMs. Workflow to obtain disease information of each virus using three LLMs from OpenAI, Google and Meta AI, and incorporating the data into respective databases. The web interface of the Viral Diseases Explorer tool (Fig. 1) accesses the information from these databases.

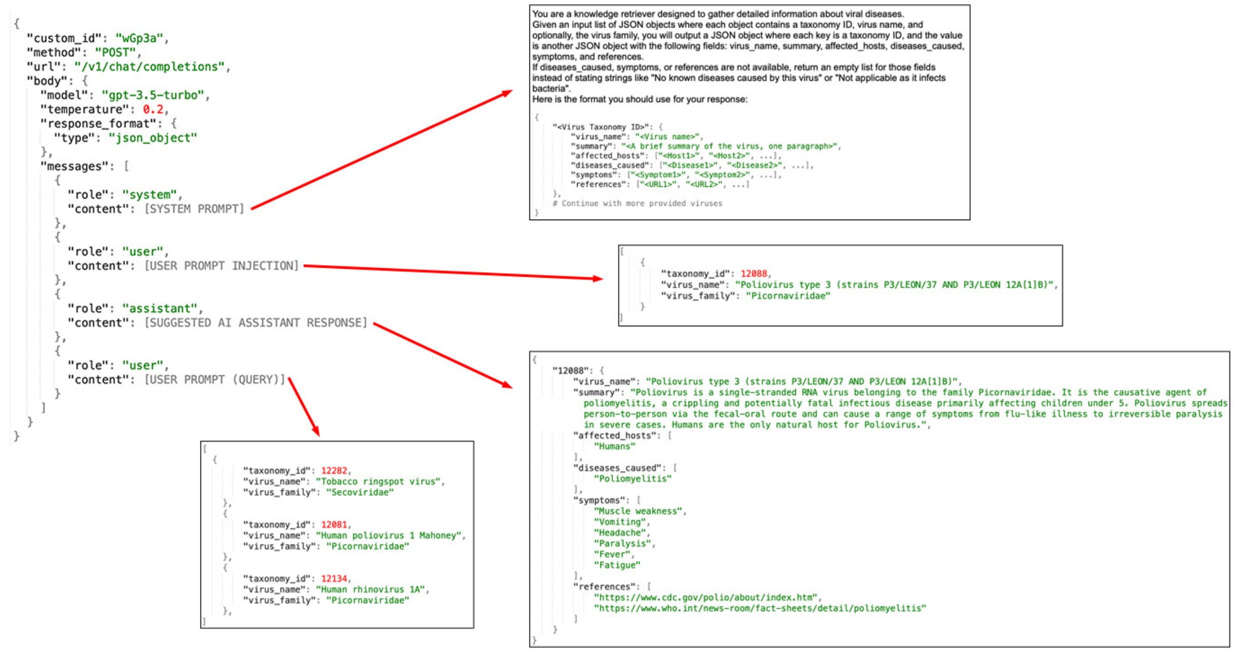

Supplementary Figure 3. An example user query submitted to OpenAI. The user query request, a JSON object, showing the various prompts - system prompt, user injection prompt, suggested/expected assistant response and lastly the actual query request - shown as insets. The same prompts were used in querying the other LLMs with some modifications to API calls.

```
{
    "3052301": {
        "virus_name": "Mammarenavirus cameroonense",
        "summary": "Mammarenavirus cameroonense is a virus belonging to the family Arenaviridae. It is named after the region of Cameroon where it
          was first identified. This virus is primarily transmitted to humans through contact with infected rodents, particularly Mastomys natalensis
          . Mammarenavirus cameroonense can cause a febrile illness known as Lassa fever, which can lead to severe hemorrhagic symptoms and multi
          -organ failure.",
        "affected_hosts": [
            "Humans",
            "Rodents (Mastomys natalensis)"
        ],
        "diseases_caused": [
            "Lassa fever"
        ],
        "symptoms": [
            "Fever",
            "Headache",
            "Muscle aches",
            "Hemorrhage",
            "Vomiting",
            "Diarrhea"
        ],
        "references": [
            "https://www.cdc.gov/vhf/lassa/index.html",
            "https://www.who.int/news-room/fact-sheets/detail/lassa-fever"
        ]
    },
    "3052302": {
        "virus_name": "Mammarenavirus chapareense",
        "summary": "Mammarenavirus chapareense is a virus of the Arenaviridae family, first identified in the Chapare region of Bolivia. This virus
          is associated with Chapare hemorrhagic fever, a disease characterized by fever, muscle pain, and bleeding manifestations. Human-to-human
          transmission of Mammarenavirus chapareense has been reported in healthcare settings.",
        "affected_hosts": [
            "Humans"
        ],
        "diseases_caused": [
            "Chapare hemorrhagic fever"
        ],
        "symptoms": [
            "Fever",
            "Muscle pain",
            "Bleeding manifestations",
            "Headache",
            "Fatigue"
        ],
        "references": [
            "https://www.who.int/news-room/fact-sheets/detail/chapare-virus"
        ]
    },
}
```


Supplementary Figure 4. An example AI-assistant's response to the user query for two viruses.

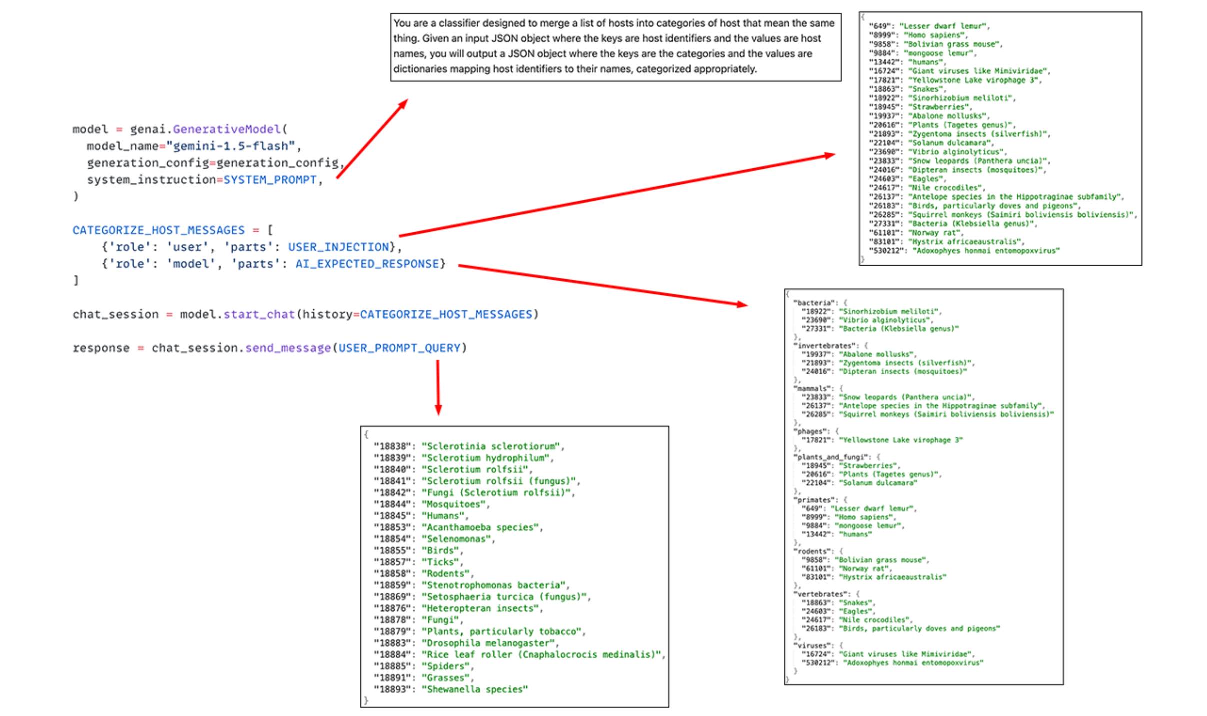

Supplementary Figure 5. An example input JSON object for classifying hostnames into various divisions using Google Gemini. Insets show the inputs for various prompts.

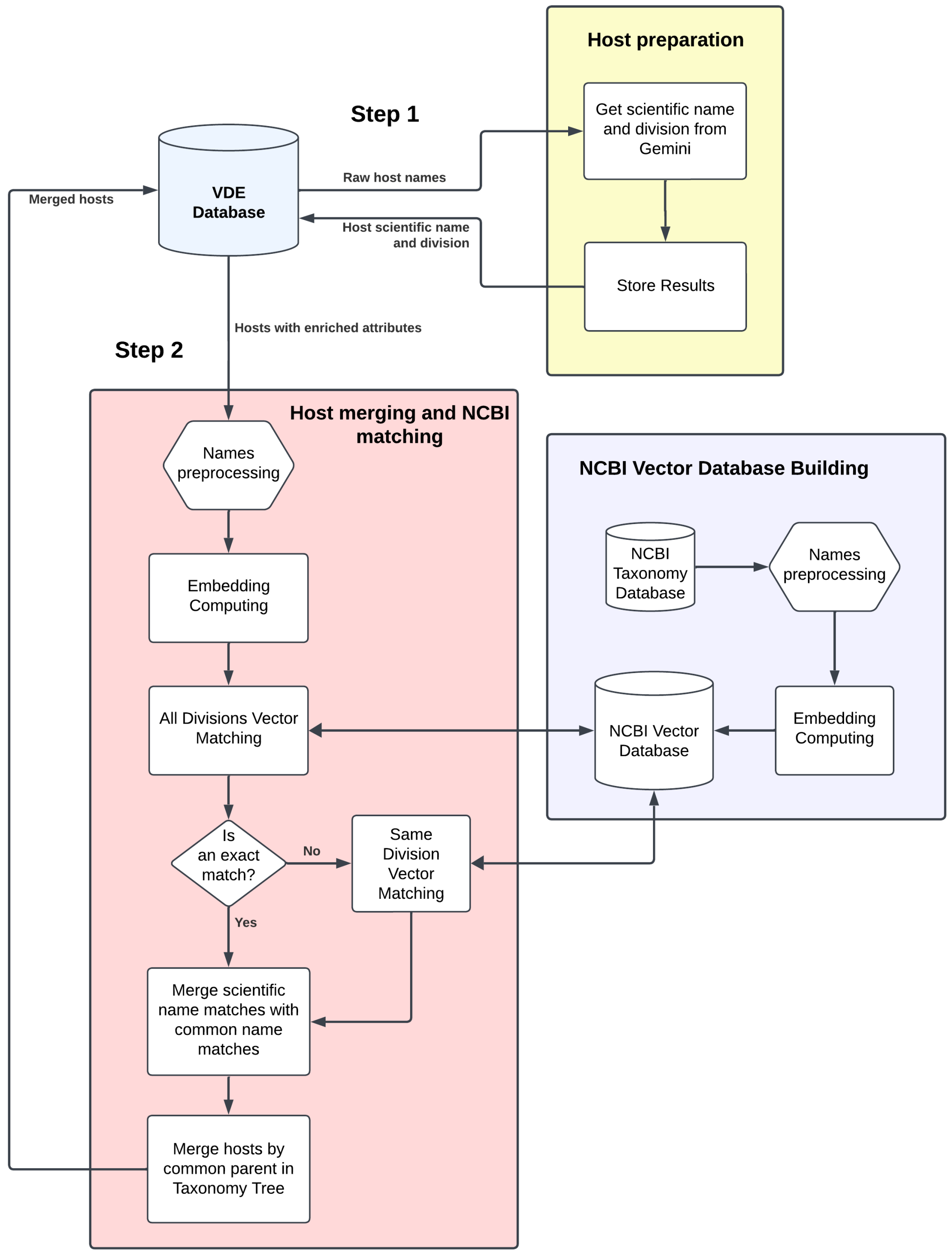

Supplementary Figure 6. Workflow for host merging and assigning NCBI:TxIDs. Step-1 involves identifying scientific names for common hostnames using Google Gemini (e.g., the scientific name for "dogs" is "Canis lupus familiaris"). Step-2 matches the scientific names with the names in the NCBI taxonomy database.

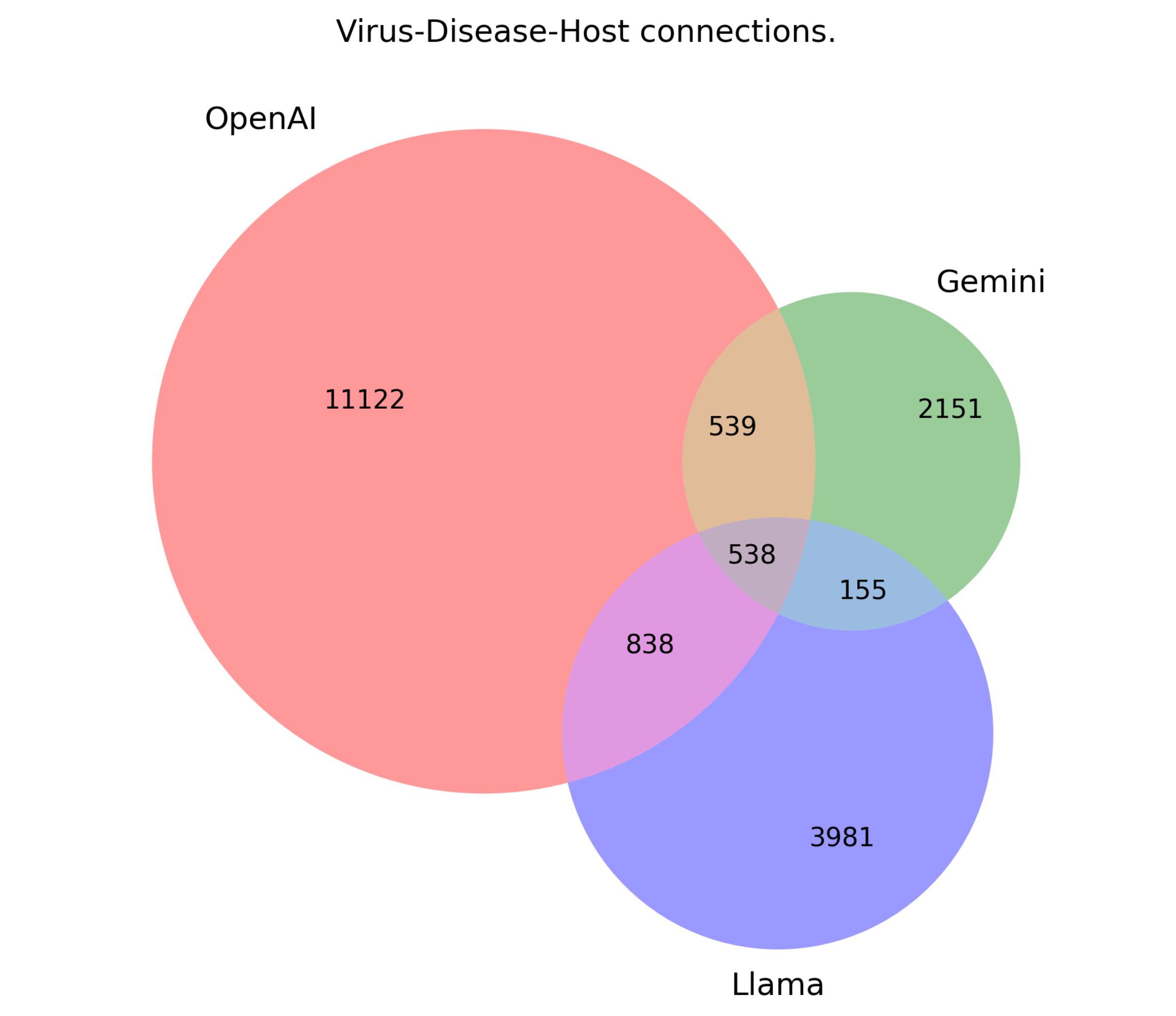

Supplementary Figure 7. Venn diagram showing the consensus virus-disease-host (tripartite) connections from the three LLMs – OpenAI GPT, Google Gemini and Mata AI's Llama.

Supplementary Table 1. Consensus virus-disease-host connections identified by the three LLMs.

Virus TxID	Virus name	Disease name	Host name	Division name
10986	Rice gall dwarf virus	Rice black gall dwarf disease	Asian cultivated rice	Plants and Fungi
2873962	Rice chlorotic stripe virus	Rice chlorotic stripe disease	Asian cultivated rice	Plants and Fungi
2910933	Rice black gall dwarf virus	Rice black gall dwarf disease	Asian cultivated rice	Plants and Fungi
2963305	Rice dwarf-associated bunya-like virus	Rice dwarf disease	Asian cultivated rice	Plants and Fungi
2760905	Domestic donkey hepatitis B virus	Hepatitis A	ass	Mammals
2802821	Asinine herpesvirus	Respiratory diseases	ass	Mammals
1110512	Balistapus sp. betanodavirus	Viral nervous necrosis	Balistapus	Vertebrates
156690	Beet mild yellowing virus	Leaf curl disease	Beta altissima	Plants and Fungi
2480685	Beet leaf yellowing virus	Leaf curl disease	Beta altissima	Plants and Fungi
11034	Sindbis virus	Sindbis fever	birds	Vertebrates
11072	Japanese encephalitis virus	Japanese encephalitis	birds	Vertebrates
11313	Fowl plague virus	Avian influenza	birds	Vertebrates
365612	Virginian WCV avipoxvirus	Avian pox	birds	Vertebrates
3016893	Avian gammacoronavirus	Infectious bronchitis	birds	Vertebrates
3078858	Urdbean crinivirus	Leaf curl disease	black gram	Plants and Fungi
3105964	Urdbean leaf crinkle disease-associated virus 1	Leaf curl disease	black gram	Plants and Fungi
536081	Blainvillea yellow spot virus	Yellow spot disease	Blainvillea	Plants and Fungi
402399	Brugmansia mild mottle virus	Leaf curl disease	Brugmansia	Plants and Fungi
2983956	Ranunculus white mottle virus_hele	Leaf curl disease	buttercups	Plants and Fungi
2056882	Cacao swollen shoot Ghana L virus	Cacao swollen shoot disease	cacao	Plants and Fungi
2057160	Cacao swollen shoot Ghana P virus	Cacao swollen shoot disease	cacao	Plants and Fungi
2057161	Cacao swollen shoot Ghana S virus	Cacao swollen shoot disease	cacao	Plants and Fungi
2057162	Cacao swollen shoot Ghana T virus	Cacao swollen shoot disease	cacao	Plants and Fungi
1027232	Groundnut ringspot and Tomato chlorotic spot virus reassortant	Chlorotic spots	Callaeum	Plants and Fungi
1027232	Groundnut ringspot and Tomato chlorotic spot virus reassortant	Groundnut ringspot	Callaeum	Plants and Fungi
2480685	Beet leaf yellowing virus	Leaf curl disease	Callaeum	Plants and Fungi
28873	Camelpox virus	Camelpox	Camelidae	Mammals
665589	Camel contagious ecthyma virus	Orf	Camelidae	Mammals
545259	Melandrium yellow fleck virus	Yellow fleck disease	campions	Plants and Fungi
337956	Bell pepper leaf curl virus	Leaf curl disease	Capsicum annuum	Plants and Fungi
3053519	Babaco ilarvirus 1	Leaf curl disease	Carica x heilbornii	Plants and Fungi
31600	Indian cassava mosaic virus	Cassava mosaic disease	cassava	Plants and Fungi

39046	Cassava common mosaic virus	Cassava mosaic disease	cassava	Plants and Fungi
62078	Cassava geminivirus	Cassava mosaic disease	cassava	Plants and Fungi
62079	East African cassava mosaic virus	Cassava mosaic disease	cassava	Plants and Fungi
137758	Cassava brown streak virus	Cassava brown streak disease	cassava	Plants and Fungi
223262	East African cassava mosaic Cameroon virus	Cassava mosaic disease	cassava	Plants and Fungi
223264	East African cassava mosaic Malawi virus	Cassava mosaic disease	cassava	Plants and Fungi
223275	East African cassava mosaic Zanzibar virus	Cassava mosaic disease	cassava	Plants and Fungi
393599	East African cassava mosaic Kenya virus	Cassava mosaic disease	cassava	Plants and Fungi
1125764	Cassava mosaic Madagascar virus	Cassava mosaic disease	cassava	Plants and Fungi
1231298	Cassava mosaic Madagascar alphasatellite	Cassava mosaic disease	cassava	Plants and Fungi
2795033	Chenopodium leaf distortion virus	Leaf curl disease	Chenopodium	Plants and Fungi
39566	Gallid herpesvirus	Infectious laryngotracheitis	chicken	Vertebrates
1566005	Chicken proventriculitis virus	Proventriculitis	chicken	Vertebrates
1162557	Chickpea yellows virus	Chickpea yellows disease	chickpea	Plants and Fungi
670548	Scomber japonicus nervous necrosis virus	Viral nervous necrosis	chub mackerel	Vertebrates
478088	Clerodendron yellow mosaic virus - [Barrackpore]	Leaf curl disease	Clerodendron	Plants and Fungi
559231	Clerodendron leaf curl virus	Leaf curl disease	Clerodendron	Plants and Fungi
2806088	Clerodendrum mosaic virus	Mosaic disease	Clerodendron	Plants and Fungi
12889	Coconut tinangaja viroid	Tinangaja disease	coconut palm	Plants and Fungi
12317	Tobacco streak virus	Tobacco streak disease	common tobacco	Plants and Fungi
67762	Tobacco leaf curl virus	Leaf curl disease	common tobacco	Plants and Fungi
205323	Tobacco leaf curl betasatellite	Leaf curl disease	common tobacco	Plants and Fungi
211866	Tobacco leaf curl Yunnan virus	Leaf curl disease	common tobacco	Plants and Fungi
223332	Tobacco leaf curl Japan virus	Leaf curl disease	common tobacco	Plants and Fungi
223334	Tobacco leaf curl Kochi virus	Leaf curl disease	common tobacco	Plants and Fungi
223337	Tobacco leaf curl Zimbabwe virus	Leaf curl disease	common tobacco	Plants and Fungi
288891	Tobacco leaf enation phytoreovirus	Leaf enation disease	common tobacco	Plants and Fungi
336987	Tobacco leaf curl Cuba virus	Leaf curl disease	common tobacco	Plants and Fungi
366679	Tobacco leaf curl Indonesia virus	Leaf curl disease	common tobacco	Plants and Fungi
409287	Tobacco leaf curl Thailand virus	Leaf curl disease	common tobacco	Plants and Fungi
439423	Tobacco leaf curl Comoros virus	Leaf curl disease	common tobacco	Plants and Fungi
530536	Tobacco mottle leaf curl virus	Leaf curl disease	common tobacco	Plants and Fungi
573594	Tobacco begomovirus - [Cuba:Granma:2007]	Leaf curl disease	common tobacco	Plants and Fungi
905054	Tobacco leaf curl Pusa virus	Leaf curl disease	common tobacco	Plants and Fungi
1213187	Tobacco leaf chlorosis betasatellite	Chlorotic and streak symptoms in plants	common tobacco	Plants and Fungi

2528965	Tobacco leaf curl Dominican Republic virus	Leaf curl disease	common tobacco	Plants and Fungi
2886212	Tobacco leaf curl Puer virus	Leaf curl disease	common tobacco	Plants and Fungi
2174956	Cowpea bright yellow mosaic virus	Leaf curl disease	cowpea	Plants and Fungi
1410062	Pumpkin yellow mosaic virus	Leaf curl disease	Cucurbita pepo	Plants and Fungi
2518373	Pumpkin polerovirus	Leaf curl disease	Cucurbita pepo	Plants and Fungi
220814	Cucurbita yellow vein virus-associated DNA beta	Yellow vein disease	Cucurbitaceae	Plants and Fungi
2974717	Paris polyphylla severe chlorotic mottle virus	Leaf curl disease	Daiswa polyphylla	Plants and Fungi
40054	Epizootic hemorrhagic disease virus	Epizootic hemorrhagic disease	deer	Mammals
104223	Deer malignant catarrhal fever virus	Malignant catarrhal fever	deer	Mammals
10785	Feline parvovirus	Feline panleukopenia	domestic cat	Mammals
10787	Feline panleukopenia virus (strain 193)	Feline panleukopenia	domestic cat	Mammals
11673	Feline immunodeficiency virus	Feline immunodeficiency virus infection	domestic cat	Mammals
11978	Feline calicivirus	Feline calicivirus infection	domestic cat	Mammals
2421386	Feline alphacoronavirus 1	Feline infectious peritonitis	domestic cat	Mammals
10243	Cowpox virus	Cowpox	domestic cattle	Mammals
11276	Vesicular stomatitis virus	Vesicular stomatitis	domestic cattle	Mammals
36370	Jembrana disease virus	Jembrana disease	domestic cattle	Mammals
129727	Bovine papular stomatitis virus	Bovine papular stomatitis	domestic cattle	Mammals
467146	Cowpox-Vaccinia virus	Cowpox	domestic cattle	Mammals
2809153	Aphthovirus sp.	Foot-and-mouth disease	domestic cattle	Mammals
1134396	Duranta erecta leaf curl Lakshmangarh virus	Leaf curl disease	Duranta plumieri	Plants and Fungi
1993907	Longan witches broom-associated virus	Witches broom disease	Euphoria longana	Plants and Fungi
2741745	Badger papillomavirus	Papillomas or warts	Eurasian badger	Mammals
664732	Castor fiber papillomavirus 1 EES-2009	Papillomas or warts	Eurasian beaver	Rodents
210022	European eel nervous necrosis virus	Viral nervous necrosis	European eel	Vertebrates
1093773	Mirabilis jalapa mottle virus	Leaf curl disease	garden four-oclock	Plants and Fungi
642478	Lettuce chlorosis virus	Lettuce chlorosis	garden lettuce	Plants and Fungi
1667233	Lettuce yellows virus	Lettuce yellows	garden lettuce	Plants and Fungi
3082270	Lactuca Big Vein associated Phlebovirus	Big vein disease	garden lettuce	Plants and Fungi
41406	Greek goat encephalitis virus	Encephalitis	goat	Mammals
89592	Brazilian caprine lentivirus	Caprine arthritis encephalitis	goat	Mammals
169959	Caprine lentivirus	Caprine arthritis encephalitis	goat	Mammals
186805	Goatpox virus	Goatpox	goat	Mammals
748171	Cyamopsis tetragonoloba leaf curl virus	Leaf curl disease	guar	Plants and Fungi

1134395	Cyamopsis tetragonoloba leaf curl Sikar virus	Leaf curl disease	guar	Plants and Fungi
28300	Heron hepatitis B virus	Hepatitis A	herons	Vertebrates
120168	Bhendi yellow vein mosaic virus	Yellow vein mosaic disease	Hibiscus esculentus	Plants and Fungi
175848	Okra yellow mottle virus	Leaf curl disease	Hibiscus esculentus	Plants and Fungi
297255	Okra yellow mottle Iguala virus	Leaf curl disease	Hibiscus esculentus	Plants and Fungi
619599	Okra mottle virus	Leaf curl disease	Hibiscus esculentus	Plants and Fungi
756758	Okra leaf curl Bihar virus	Okra leaf curl disease	Hibiscus esculentus	Plants and Fungi
759124	Bhendi yellow vein mosaic alphasatellite	Yellow vein mosaic disease	Hibiscus esculentus	Plants and Fungi
908070	Bhendi yellow vein mosaic betasatellite	Yellow vein mosaic disease	Hibiscus esculentus	Plants and Fungi
912034	Okra leaf curl Cameroon virus	Okra leaf curl disease	Hibiscus esculentus	Plants and Fungi
1225644	Bhendi yellow vein mosaic virus-associated alphasatellite	Yellow vein mosaic disease	Hibiscus esculentus	Plants and Fungi
2010323	Okra leaf curl Oman betasatellite	Okra leaf curl disease	Hibiscus esculentus	Plants and Fungi
199587	Hollyhock leaf crumple virus	Leaf crumple disease	hollyhock	Plants and Fungi
944994	Hollyhock yellow vein mosaic virus	Yellow vein mosaic disease	hollyhock	Plants and Fungi
2231665	Hollyhock leaf curl alphasatellite	Leaf curl disease	hollyhock	Plants and Fungi
89463	Sacbrood virus	Sacbrood disease	honey bee	Invertebrates
458132	Slow bee paralysis virus	Bee paralysis	honey bee	Invertebrates
12327	Barley stripe mosaic virus	Leaf curl disease	Hordeum vulgare	Plants and Fungi
12465	Barley yellow mosaic virus	Leaf curl disease	Hordeum vulgare	Plants and Fungi
100887	Soil-borne cereal mosaic virus	Soil-borne cereal mosaic	Hordeum vulgare	Plants and Fungi
2169981	Barley yellow dwarf virus SGV	Yellow dwarf disease	Hordeum vulgare	Plants and Fungi
11037	Venezuelan equine encephalitis virus (strain TC-83)	Venezuelan equine encephalitis	horse	Mammals
11039	Western equine encephalitis virus	Equine encephalitis	horse	Mammals
11276	Vesicular stomatitis virus	Vesicular stomatitis	horse	Mammals
11665	Equine infectious anemia virus	Equine infectious anemia	horse	Mammals
12455	Borna disease virus	Borna disease	horse	Mammals
47000	Equine rhinitis A virus	Equine rhinitis	horse	Mammals
201490	Equine encephalosis virus	Equine encephalitis	horse	Mammals
356862	Peruvian horse sickness virus	Peruvian horse sickness	horse	Mammals
2025828	Equine papillomavirus	Papillomas or warts	horse	Mammals
2499620	Alphaarterivirus equid	Equine viral arteritis	horse	Mammals
10243	Cowpox virus	Cowpox	human	Primates
10244	Monkeypox virus	Monkeypox	human	Primates
10245	Vaccinia virus	Vaccinia	human	Primates
10255	Variola virus	Smallpox	human	Primates

10279	Molluscum contagiosum virus	Molluscum contagiosum	human	Primates
10368	Human betaherpesvirus 6	Encephalitis	human	Primates
11033	Semliki Forest virus	Encephalitis	human	Primates
11034	Sindbis virus	Sindbis fever	human	Primates
11037	Venezuelan equine encephalitis virus (strain TC-83)	Venezuelan equine encephalitis	human	Primates
11039	Western equine encephalitis virus	Equine encephalitis	human	Primates
11072	Japanese encephalitis virus	Japanese encephalitis	human	Primates
11087	Tick-borne encephalitis virus (STRAIN SOFJIN)	Tick-borne encephalitis	human	Primates
11966	Aids-associated retrovirus	Acquired immunodeficiency syndrome (AIDS)	human	Primates
11983	Norwalk virus	Gastroenteritis	human	Primates
12098	Human hepatitis A virus Hu/Australia/HM175/1976	Hepatitis A	human	Primates
12475	Hepatitis delta virus	Hepatitis D	human	Primates
33774	Hepatitis E virus (strain Pakistan)	Hepatitis E	human	Primates
37296	Human gammaherpesvirus 8	Kaposi's sarcoma	human	Primates
39137	Tick-borne flavivirus	Tick-borne encephalitis	human	Primates
42097	Isla Vista hantavirus	Hantavirus Pulmonary Syndrome	human	Primates
47301	Muleshoe hantavirus	Hantavirus Pulmonary Syndrome	human	Primates
64316	Russian Spring-Summer encephalitis virus	Russian Spring-Summer encephalitis	human	Primates
77763	Banna virus	Febrile illness	human	Primates
95342	Sapporo virus	Gastroenteritis	human	Primates
99000	Tanapox virus	Tanapox	human	Primates
118043	Untyped human rhinovirus OK88-8162	Common cold	human	Primates
178534	JC virus x BK virus	Progressive multifocal leukoencephalopathy (PML)	human	Primates
205488	Ebola virus sp.	Ebola virus disease	human	Primates
224649	Oyster norovirus	Gastroenteritis	human	Primates
235406	untyped human enterovirus	Meningitis	human	Primates
261202	Alto Paraguay hantavirus	Hantavirus Pulmonary Syndrome	human	Primates
315431	untyped Human adenovirus	Conjunctivitis	human	Primates
318275	Untyped Human T-lymphotropic virus	Adult T-cell leukemia/lymphoma	human	Primates
318275	Untyped Human T-lymphotropic virus	HTLV-1-associated myelopathy/tropical spastic paraparesis	human	Primates
374990	Mayaro virus (strain Brazil)	Mayaro fever	human	Primates
430511	Juquitiba virus	Hantavirus Pulmonary Syndrome	human	Primates
467146	Cowpox-Vaccinia virus	Cowpox	human	Primates

493803	Merkel cell polyomavirus	Merkel cell carcinoma	human	Primates
650132	Aichi virus A846/88	Gastroenteritis	human	Primates
1095764	Caliciviridae Hu/GII.4/Xiamen/May0511/2010/CHN	Gastroenteritis	human	Primates
1244521	ANAJ Hantavirus	Hantavirus Pulmonary Syndrome	human	Primates
1244522	CASV Hantavirus	Hantavirus Pulmonary Syndrome	human	Primates
1244523	CASV-2 Hantavirus	Hantavirus Pulmonary Syndrome	human	Primates
1314973	Lianghe virus	Hemorrhagic Fever with Renal Syndrome (HFRS)	human	Primates
1518574	Burkina Faso astrovirus	Gastroenteritis	human	Primates
1933189	Sandfly fever Naples phlebovirus	Sandfly fever	human	Primates
1933190	SFTS phlebovirus	Severe Fever with Thrombocytopenia Syndrome (SFTS)	human	Primates
2169701	Onyong-nyong virus	Onyong-nyong fever	human	Primates
2304450	Dakrong virus	Hantavirus Pulmonary Syndrome	human	Primates
2558248	Adenoviridae sp.	Conjunctivitis	human	Primates
2748762	Colorado tick fever coltivirus	Colorado tick fever	human	Primates
2778957	Biya River virus	Hemorrhagic Fever with Renal Syndrome (HFRS)	human	Primates
2994337	Human hepatitis E virus genotype 3	Hepatitis E	human	Primates
3040071	Human orthopneumovirus subgroup A	Bronchiolitis	human	Primates
3228684	Sager Creek orthohantavirus	Hantavirus Pulmonary Syndrome	human	Primates
375546	Hydrangea chlorotic mottle virus	Leaf curl disease	Hydrangea	Plants and Fungi
1367183	Hypericum yellow mosaic virus	Leaf curl disease	Hypericum	Plants and Fungi
3035954	Prunus mume chlorotic leaf curl-associated virus	Leaf curl disease	Japanese apricot	Plants and Fungi
394239	Koala retrovirus	Leukemia	koala	Mammals
394239	Koala retrovirus	Lymphoma	koala	Mammals
10839	Bean golden mosaic virus	Leaf curl disease	Leguminosae	Plants and Fungi
57119	Bean yellow dwarf virus	Leaf curl disease	Leguminosae	Plants and Fungi
714310	Bean yellow mosaic Mexico virus	Leaf curl disease	Leguminosae	Plants and Fungi
207240	Luffa yellow mosaic virus	Leaf curl disease	Luffa	Plants and Fungi
333675	Luffa begomovirus	Leaf curl disease	Luffa	Plants and Fungi
1134398	Luffa cylindrica yellow mosaic Bhatinda virus	Leaf curl disease	Luffa sphaerica	Plants and Fungi
335484	Malvastrum coromandelianum yellow vein virus	Yellow vein disease	Malva coromandeliana	Plants and Fungi
1170660	Marigold leaf curl virus	Leaf curl disease	marigolds	Plants and Fungi
126997	Marmoset hepatitis GB virus A	Hepatitis A	marmosets	Primates
10829	Squash leaf curl virus	Leaf curl disease	marrows	Plants and Fungi
91372	Squash yellow mottle virus	Leaf curl disease	marrows	Plants and Fungi

220742	Squash leaf curl Israel virus	Leaf curl disease	marrows	Plants and Fungi
223323	Squash leaf curl China virus	Leaf curl disease	marrows	Plants and Fungi
223324	Squash mild leaf curl virus	Leaf curl disease	marrows	Plants and Fungi
1051966	Squash yellow mild mottle virus	Leaf curl disease	marrows	Plants and Fungi
2419504	Squash leaf curl Thailand virus	Leaf curl disease	marrows	Plants and Fungi
1170655	Melia leaf curl virus	Leaf curl disease	Melia azedarach	Plants and Fungi
1742594	Melochia mosaic virus	Mosaic disease	Melochia	Plants and Fungi
39137	Tick-borne flavivirus	Tick-borne encephalitis	metazoans	Invertebrates
667119	Velvet bean severe mosaic virus	Mosaic disease	Mucuna prurita	Plants and Fungi
1881630	Velvet bean golden mosaic virus	Golden mosaic disease	Mucuna prurita	Plants and Fungi
165826	Melon chlorotic leaf curl virus	Leaf curl disease	muskmelon	Plants and Fungi
180399	Melon chlorotic mosaic virus	Leaf curl disease	muskmelon	Plants and Fungi
1419730	Melon begomovirus	Leaf curl disease	muskmelon	Plants and Fungi
1602199	Oxalis yellow vein virus	Yellow vein disease	Oxalis	Plants and Fungi
53260	Papaya leaf curl virus	Leaf curl disease	papaya	Plants and Fungi
229634	Papaya leaf curl China virus	Leaf curl disease	papaya	Plants and Fungi
271479	Papaya leaf curl Guandong virus	Leaf curl disease	papaya	Plants and Fungi
447599	Papaya leaf curl China betasatellite	Leaf curl disease	papaya	Plants and Fungi
453453	Papaya leaf curl beta - [India:Chinthapalli:2005]	Leaf curl disease	papaya	Plants and Fungi
714640	Papaya leaf curl betasatellite	Leaf curl disease	papaya	Plants and Fungi
1504733	Papaya leaf curl Faisalabad virus	Leaf curl disease	papaya	Plants and Fungi
1939619	Papaya yellow leaf curl virus	Leaf curl disease	papaya	Plants and Fungi
2010324	Papaya leaf curl India betasatellite	Leaf curl disease	papaya	Plants and Fungi
2876733	Papaya leaf curl Guntur virus	Leaf curl disease	papaya	Plants and Fungi
2909202	Papaya leaf curl Vikaspuri virus	Leaf curl disease	papaya	Plants and Fungi
2978335	Papaya leaf curl distortion virus	Leaf curl disease	papaya	Plants and Fungi
2978336	Papaya mottle leaf curl virus	Leaf curl disease	papaya	Plants and Fungi
3002632	Papaya leaf curl Faridabad betasatellite	Leaf curl disease	papaya	Plants and Fungi
3019450	Papaya leaf curl Bagalkote virus	Leaf curl disease	papaya	Plants and Fungi
3019451	Papaya leaf curl Haveri virus	Leaf curl disease	papaya	Plants and Fungi
3020611	Papaya leaf curl Vikaspuri alphasatellite	Leaf curl disease	papaya	Plants and Fungi
3114929	Papaya sticky fruit-associated virus	Sticky fruit disease	papaya	Plants and Fungi
3133020	Papaya leaf curl distortion deltasatellite 1	Leaf curl disease	papaya	Plants and Fungi
3145073	papaya leaf curl Lucknow betasatellite	Leaf curl disease	papaya	Plants and Fungi
77856	Beak and feather disease virus	Beak and feather disease	parrots and others	Vertebrates
2683337	Parsley yellow leaf curl virus	Leaf curl disease	parsley	Plants and Fungi
12894	Peach latent mosaic viroid	Peach latent mosaic disease	peach	Plants and Fungi
471498	Peach chlorotic mottle virus	Leaf curl disease	peach	Plants and Fungi
28355	Peanut clump virus	Peanut clump disease	peanut	Plants and Fungi

60596	Peanut chlorotic blotch virus	Chlorotic blotch	peanut	Plants and Fungi
80271	Peanut chlorotic fan-spot virus	Chlorotic fan-spot disease	peanut	Plants and Fungi
12239	Pepper mild mottle virus	Leaf curl disease	peppers	Plants and Fungi
31750	Pepper ringspot virus	Ringspot disease	peppers	Plants and Fungi
83839	Pepper leaf curl virus	Leaf curl disease	peppers	Plants and Fungi
341745	Chili leaf curl Bangladesh virus	Leaf curl disease	peppers	Plants and Fungi
1003836	Pepper leaf curl Yunnan virus	Leaf curl disease	peppers	Plants and Fungi
1387160	Pepper leafroll virus	Chlorotic and streak symptoms in plants	peppers	Plants and Fungi
1507724	Chilli leaf curl Dhanbad alphasatellite	Leaf curl disease	peppers	Plants and Fungi
1507725	Chilli leaf curl India alphasatellite	Leaf curl disease	peppers	Plants and Fungi
1701401	Chilli leaf curl Bijnour virus-India	Leaf curl disease	peppers	Plants and Fungi
2050929	Chilli leaf curl Vellanad virus	Leaf curl disease	peppers	Plants and Fungi
2050930	Chilli leaf curl Kanpur virus	Leaf curl disease	peppers	Plants and Fungi
2052172	Pepper leafroll chlorosis virus	Chlorotic and streak symptoms in plants	peppers	Plants and Fungi
2560381	Chilli leaf curl Sri Lanka virus	Leaf curl disease	peppers	Plants and Fungi
2766545	Chilli leaf curl Coochbehar virus	Leaf curl disease	peppers	Plants and Fungi
2792284	Chili pepper mild mottle virus	Leaf curl disease	peppers	Plants and Fungi
2839820	Chilli leaf curl Guntur virus	Leaf curl disease	peppers	Plants and Fungi
2975233	Chilli leaf curl Nagpur virus	Leaf curl disease	peppers	Plants and Fungi
2978733	Chilli leaf curl Ahmedabad alphasatellite	Leaf curl disease	peppers	Plants and Fungi
3029073	Chilli leaf curl Pakistan alphasatellite	Leaf curl disease	peppers	Plants and Fungi
500654	Bandicoot papillomatosis carcinomatosis virus type 2	Papillomas or warts	Peramelemorphia	Mammals
1218727	French bean severe leaf curl virus	Leaf curl disease	Phaseolus vulgaris	Plants and Fungi
2010320	French bean leaf curl betasatellite	Leaf curl disease	Phaseolus vulgaris	Plants and Fungi
2050584	French bean leaf curl virus	Leaf curl disease	Phaseolus vulgaris	Plants and Fungi
2849243	French bean leaf curl Madikeri virus	Leaf curl disease	Phaseolus vulgaris	Plants and Fungi
2885928	Physalis vein necrosis virus	Vein necrosis	Physalis	Plants and Fungi
3024159	Physalis chlorosis virus	Chlorosis	Physalis	Plants and Fungi
2987725	Physalis minima leaf curl betsatellite	Leaf curl disease	Physalis minima	Plants and Fungi
10276	Swinepox virus	Swinepox	pig	Mammals
10497	African swine fever virus	African swine fever	pig	Mammals
11072	Japanese encephalitis virus	Japanese encephalitis	pig	Mammals
11276	Vesicular stomatitis virus	Vesicular stomatitis	pig	Mammals
12845	Swine influenza virus	Swine influenza	pig	Mammals
28295	Porcine epidemic diarrhea virus	Porcine epidemic diarrhea	pig	Mammals
33774	Hepatitis E virus (strain Pakistan)	Hepatitis E	pig	Mammals

942031	Circoviridae PorkNW2/USA/2009	Porcine circovirus-associated disease	pig	Mammals
2809153	Aphthovirus sp.	Foot-and-mouth disease	pig	Mammals
2978516	Porcine coronavirus	Porcine epidemic diarrhea	pig	Mammals
156441	Pigeon pea yellow mosaic virus	Leaf curl disease	pigeon pea	Plants and Fungi
2152757	Cajanus cajan yellow mosaic virus	Leaf curl disease	pigeon pea	Plants and Fungi
28354	Plantago asiatica mosaic virus	Mosaic disease	Plantago asiatica	Plants and Fungi
10827	Potato yellow mosaic virus	Leaf curl disease	potato	Plants and Fungi
215340	Potato apical leaf curl virus	Leaf curl disease	potato	Plants and Fungi
223307	Potato yellow mosaic Panama virus	Leaf curl disease	potato	Plants and Fungi
223308	Potato yellow mosaic Trinidad virus	Leaf curl disease	potato	Plants and Fungi
410826	Potato apical leaf curl disease-associated satellite DNA beta	Leaf curl disease	potato	Plants and Fungi
37477	Prairie vole hantavirus	Hantavirus Pulmonary Syndrome	prairie vole	Rodents
10244	Monkeypox virus	Monkeypox	primates	Primates
205488	Ebola virus sp.	Ebola virus disease	primates	Primates
444185	Simian rotavirus A strain RRV	Gastroenteritis	primates	Primates
1903318	Primate norovirus	Gastroenteritis	primates	Primates
2482770	Pseudodiaptomus annandalei nervous necrosis virus	Nervous necrosis	Pseudodiaptomus annandalei	Invertebrates
10273	Myxoma virus	Myxomatosis	rabbit	Mammals
1038051	Rabbit adenovirus Wisconsin	Respiratory diseases	rabbit	Mammals
1889900	Rabbit calicivirus Australia 1	Rabbit hemorrhagic disease	rabbit	Mammals
2792258	Raccoon dog acute diarrhea syndrome coronavirus	Diarrhea	raccoon dog	Mammals
662596	Radish leaf curl betasatellite	Leaf curl disease	radish	Plants and Fungi
1507726	Radish leaf curl alphasatellite	Leaf curl disease	radish	Plants and Fungi
31695	Rat leukemia virus	Leukemia	Rattus	Rodents
210025	Hump-back grouper nervous necrosis virus	Viral nervous necrosis	ray-finned fishes	Vertebrates
11033	Semliki Forest virus	Encephalitis	rodent	Rodents
42097	Isla Vista hantavirus	Hantavirus Pulmonary Syndrome	rodent	Rodents
47301	Muleshoe hantavirus	Hantavirus Pulmonary Syndrome	rodent	Rodents
261202	Alto Paraguay hantavirus	Hantavirus Pulmonary Syndrome	rodent	Rodents
286540	Soochong virus	Hemorrhagic Fever with Renal Syndrome (HFRS)	rodent	Rodents
430511	Juquitiba virus	Hantavirus Pulmonary Syndrome	rodent	Rodents
937388	Dahonggou Creek virus	Hantavirus Pulmonary Syndrome	rodent	Rodents
1107325	Ussuri virus	Hemorrhagic Fever with Renal Syndrome (HFRS)	rodent	Rodents

1201042	Kilimanjaro virus	Hantavirus Pulmonary Syndrome	rodent	Rodents
1244521	ANAJ Hantavirus	Hantavirus Pulmonary Syndrome	rodent	Rodents
1244525	LANV-2 Hantavirus	Hantavirus Pulmonary Syndrome	rodent	Rodents
1314972	Huangpi virus	Hemorrhagic Fever with Renal Syndrome (HFRS)	rodent	Rodents
1314973	Lianghe virus	Hemorrhagic Fever with Renal Syndrome (HFRS)	rodent	Rodents
1538453	Yuanjiang virus	Hantavirus Pulmonary Syndrome	rodent	Rodents
2050013	Rodent hantavirus	Hantavirus Pulmonary Syndrome	rodent	Rodents
2050018	Rodent coronavirus	Respiratory diseases	rodent	Rodents
2137546	Rodent bocavirus	Respiratory diseases	rodent	Rodents
2259728	Monongahela hantavirus	Hantavirus Pulmonary Syndrome	rodent	Rodents
2304450	Dakrong virus	Hantavirus Pulmonary Syndrome	rodent	Rodents
2778957	Biya River virus	Hemorrhagic Fever with Renal Syndrome (HFRS)	rodent	Rodents
2809448	Hantaviridae sp.	Hantavirus Pulmonary Syndrome	rodent	Rodents
2809448	Hantaviridae sp.	Hemorrhagic Fever with Renal Syndrome (HFRS)	rodent	Rodents
3127671	Ozark orthohantavirus	Hantavirus Pulmonary Syndrome	rodent	Rodents
3228684	Sager Creek orthohantavirus	Hantavirus Pulmonary Syndrome	rodent	Rodents
1048434	Rose yellow mosaic virus	Rose yellow mosaic disease	Rosa	Plants and Fungi
11005	Infectious pancreatic necrosis virus - Sp	Infectious pancreatic necrosis	salmonids	Vertebrates
55987	Isavirus salaris	Infectious salmon anemia	salmonids	Vertebrates
1130981	Sauropus leaf curl virus	Leaf curl disease	Sauropus	Plants and Fungi
10266	Sheeppox virus	Sheeppox	sheep	Mammals
11746	Jaagsiekte sheep retrovirus	Ovine pulmonary adenocarcinoma	sheep	Mammals
12455	Borna disease virus	Borna disease	sheep	Mammals
40541	Ovine papillomavirus	Papillomas or warts	sheep	Mammals
41408	Spanish sheep encephalitis virus	Encephalitis	sheep	Mammals
50359	Ovine pestivirus	Border disease	sheep	Mammals
253923	Ovine progressive pneumonia virus	Ovine progressive pneumonia	sheep	Mammals
2169971	Visna-maedi virus	Visna	sheep	Mammals
2169971	Visna-maedi virus	Maedi	sheep	Mammals
2809153	Aphthovirus sp.	Foot-and-mouth disease	sheep	Mammals
223317	Sida golden yellow vein virus	Yellow vein disease	Sida	Invertebrates
337823	Sida leaf curl virus	Leaf curl disease	Sida	Invertebrates

420255	Sida yellow vein Madurai virus	Yellow vein disease	Sida	Invertebrates
536083	Sida yellow leaf curl virus	Leaf curl disease	Sida	Invertebrates
759390	Sida golden mottle virus	Sida golden mottle disease	Sida	Invertebrates
858513	Sidastrum golden leaf spot virus	Chlorotic spots	Sidastrum	Plants and Fungi
85753	Soybean crinkle leaf virus	Leaf curl disease	soybean	Plants and Fungi
761702	Soybean chlorotic blotch virus	Chlorotic blotch	soybean	Plants and Fungi
1221206	Soybean chlorotic spot virus	Chlorotic spots	soybean	Plants and Fungi
2831441	Soybean yellow dwarf-associated geminivirus	Yellow dwarf disease	soybean	Plants and Fungi
2951633	Soybean yellow leaf curl virus	Leaf curl disease	soybean	Plants and Fungi
2959664	Soybean chlorotic leafroll virus	Chlorotic and streak symptoms in plants	soybean	Plants and Fungi
873160	Spinach severe curly top virus	Curly top disease	spinach	Plants and Fungi
65467	Cherry green ring mottle virus	Ring mottle disease	sweet cherry	Plants and Fungi
284688	Cherry chlorotic rusty spot associated partitivirus	Cherry rusty mottle disease	sweet cherry	Plants and Fungi
1312929	Cherry rusty mottle associated virus	Cherry rusty mottle disease	sweet cherry	Plants and Fungi
100755	Sweet potato leaf curl virus	Leaf curl disease	sweet potato	Plants and Fungi
225752	Sweet potato leaf curl Georgia virus	Leaf curl disease	sweet potato	Plants and Fungi
574890	Sweet potato leaf curl Lanzarote virus	Leaf curl disease	sweet potato	Plants and Fungi
652717	Sweet potato leaf curl Spain virus	Leaf curl disease	sweet potato	Plants and Fungi
673321	Sweetpotato leaf curl virus	Leaf curl disease	sweet potato	Plants and Fungi
693892	Sweet potato leaf curl Bengal virus	Leaf curl disease	sweet potato	Plants and Fungi
994388	Sweet potato leaf curl Sao Paulo virus	Leaf curl disease	sweet potato	Plants and Fungi
999288	Sweet potato leaf curl South Carolina virus	Leaf curl disease	sweet potato	Plants and Fungi
1198219	Sweet potato leaf curl China virus	Leaf curl disease	sweet potato	Plants and Fungi
1350389	Sweet potato leaf curl China Henan virus	Leaf curl disease	sweet potato	Plants and Fungi
1392387	Sweet potato leaf curl Japan virus	Leaf curl disease	sweet potato	Plants and Fungi
1392388	Sweet potato leaf curl Shanghai virus	Leaf curl disease	sweet potato	Plants and Fungi
1489311	Sweet potato leaf curl Henan virus	Leaf curl disease	sweet potato	Plants and Fungi
1489312	Sweet potato leaf curl Guangxi virus	Leaf curl disease	sweet potato	Plants and Fungi
1513527	Sweet potato leaf curl Uganda virus	Leaf curl disease	sweet potato	Plants and Fungi
1827003	Sweet potato leaf curl Henanzhengzhou virus	Leaf curl disease	sweet potato	Plants and Fungi
1861791	Sweet potato leaf curl Jiangsu3 virus	Leaf curl disease	sweet potato	Plants and Fungi
2026195	Sweet potato leaf curl Sichuan virus 2	Leaf curl disease	sweet potato	Plants and Fungi
2583405	Sweet potato leaf curl Shandong virus 2	Leaf curl disease	sweet potato	Plants and Fungi
2892858	Leaf curl disease	sweet potato	Plants and Fungi	
10832	Tomato yellow leaf curl virus	Leaf curl disease	tomato	Plants and Fungi
10835	Tomato mottle virus	Leaf curl disease	tomato	Plants and Fungi

28350	Tomato leaf curl virus	Leaf curl disease	tomato	Plants and Fungi
53481	Tomato leaf curl Tanzania virus	Leaf curl disease	tomato	Plants and Fungi
53991	Tomato leaf curl Java betasatellite	Leaf curl disease	tomato	Plants and Fungi
67754	Tomato chlorosis virus	Tomato chlorosis disease	tomato	Plants and Fungi
70696	Jamaican tomato infecting geminivirus	Leaf curl disease	tomato	Plants and Fungi
71186	Tomato leaf curl Sinaloa virus	Leaf curl disease	tomato	Plants and Fungi
85752	Tomato yellow leaf curl Thailand virus	Leaf curl disease	tomato	Plants and Fungi
86502	Tomato yellow mottle virus	Leaf curl disease	tomato	Plants and Fungi
92943	Tomato mild yellow mottle virus - [Honduras 96 - H5kw]	Leaf curl disease	tomato	Plants and Fungi
92963	Tomato severe leaf curl virus	Leaf curl disease	tomato	Plants and Fungi
96054	Tomato leaf curl Philippines virus	Leaf curl disease	tomato	Plants and Fungi
123735	Tomato yellow leaf curl Sardinia virus	Leaf curl disease	tomato	Plants and Fungi
158463	Tomato severe rugose virus	Tomato severe rugose disease	tomato	Plants and Fungi
172390	Tomato chlorotic vein virus	Chlorotic and streak symptoms in plants	tomato	Plants and Fungi
172391	Tomato chlorotic mottle virus	Leaf curl disease	tomato	Plants and Fungi
172394	Tomato mottle leaf curl virus	Leaf curl disease	tomato	Plants and Fungi
178599	Tomato mild mottle virus	Leaf curl disease	tomato	Plants and Fungi
185793	Tomato yellow leaf curl China virus	Leaf curl disease	tomato	Plants and Fungi
191461	Tomato crinkle yellow leaf virus	Leaf curl disease	tomato	Plants and Fungi
196093	Tomato leaf curl Taiwan virus	Leaf curl disease	tomato	Plants and Fungi
200392	Tomato yellow fruit ring virus	Tomato yellow fruit ring disease	tomato	Plants and Fungi
208177	Tomato leaf curl Vietnam virus	Leaf curl disease	tomato	Plants and Fungi
219299	Tomato leaf curl Gujarat virus	Leaf curl disease	tomato	Plants and Fungi
219744	Tomato yellow leaf curl Malaga virus	Leaf curl disease	tomato	Plants and Fungi
223344	Tomato leaf curl Bangladesh virus	Leaf curl disease	tomato	Plants and Fungi
223345	Tomato leaf curl Laos virus	Leaf curl disease	tomato	Plants and Fungi
223346	Tomato leaf curl Malaysia virus	Leaf curl disease	tomato	Plants and Fungi
223352	Tomato leaf curl Sri Lanka virus	Leaf curl disease	tomato	Plants and Fungi
223356	Tomato mosaic Havana virus	Leaf curl disease	tomato	Plants and Fungi
223358	Tomato mottle Taino virus	Leaf curl disease	tomato	Plants and Fungi
232380	Tomato yellow leaf curl Axarquia virus	Leaf curl disease	tomato	Plants and Fungi
240492	Tomato chino La Paz virus	Leaf curl disease	tomato	Plants and Fungi
240557	Tomato yellow leaf curl China alphasatellite	Leaf curl disease	tomato	Plants and Fungi
260377	Tomato yellow leaf curl Mali virus	Leaf curl disease	tomato	Plants and Fungi
260379	Tomato leaf curl Mali virus	Leaf curl disease	tomato	Plants and Fungi
263001	Tomato leaf curl Java virus	Leaf curl disease	tomato	Plants and Fungi
266799	Tomato yellow leaf curl Kanchanaburi virus	Leaf curl disease	tomato	Plants and Fungi
270140	Tomato leaf curl Iran virus	Leaf curl disease	tomato	Plants and Fungi

270146	Tomato leaf curl Sudan virus	Leaf curl disease	tomato	Plants and Fungi
278069	Tomato leaf curl China betasatellite	Leaf curl disease	tomato	Plants and Fungi
294828	Tomato leaf curl Las Playitas virus	Leaf curl disease	tomato	Plants and Fungi
297249	Tomato yellow leaf curl Guangdong virus	Leaf curl disease	tomato	Plants and Fungi
302100	Tomato leaf curl Mayotte virus	Leaf curl disease	tomato	Plants and Fungi
341034	Tomato leaf curl Uganda virus	Leaf curl disease	tomato	Plants and Fungi
341711	Tomato leaf curl Pakistan virus	Leaf curl disease	tomato	Plants and Fungi
366920	Tomato mild leaf curl virus - [Uberlandia]	Leaf curl disease	tomato	Plants and Fungi
377609	Tomato leaf curl Guangxi virus	Leaf curl disease	tomato	Plants and Fungi
390442	Tomato yellow leaf curl Vietnam virus	Leaf curl disease	tomato	Plants and Fungi
399387	Tomato leaf curl Hsinchu virus	Leaf curl disease	tomato	Plants and Fungi
400835	Tomato leaf curl Guangdong virus	Leaf curl disease	tomato	Plants and Fungi
406088	Tomato leaf curl Dharwad virus	Leaf curl disease	tomato	Plants and Fungi
407837	Tomato leaf curl Pune virus	Leaf curl disease	tomato	Plants and Fungi
420253	Tomato leaf curl Rajasthan virus	Leaf curl disease	tomato	Plants and Fungi
425303	Tomato leaf curl Seychelles virus	Leaf curl disease	tomato	Plants and Fungi
439425	Tomato leaf curl Moheli virus	Leaf curl disease	tomato	Plants and Fungi
439426	Tomato leaf curl Diana virus	Leaf curl disease	tomato	Plants and Fungi
439427	Tomato leaf curl Toliara virus	Leaf curl disease	tomato	Plants and Fungi
447604	Tomato yellow leaf curl Vietnam betasatellite	Leaf curl disease	tomato	Plants and Fungi
453451	Tomato leaf curl Maharastra beta - [India:Pune:2004]	Leaf curl disease	tomato	Plants and Fungi
478812	Tomato leaf curl New Delhi betasatellite	Leaf curl disease	tomato	Plants and Fungi
501328	Tomato leaf curl Ghana virus	Leaf curl disease	tomato	Plants and Fungi
507475	Tomato leaf curl Cotabato virus	Leaf curl disease	tomato	Plants and Fungi
536084	Tomato common mosaic virus	Leaf curl disease	tomato	Plants and Fungi
537918	TYLCV/TYLCSV recombinant Ragusa/2004	Leaf curl disease	tomato	Plants and Fungi
538004	Tomato leaf curl Pakistan alphasatellite	Leaf curl disease	tomato	Plants and Fungi
544743	Tomato yellow blotch virus	Yellow blotch disease	tomato	Plants and Fungi
548915	Tomato leaf curl Kumasi virus	Leaf curl disease	tomato	Plants and Fungi
551531	Tomato leaf curl Kerala virus	Leaf curl disease	tomato	Plants and Fungi
559232	Tomato leaf curl Fontem virus	Leaf curl disease	tomato	Plants and Fungi
564944	Tomato yellow distortion leaf virus	Leaf curl disease	tomato	Plants and Fungi
575917	Tomato leaf curl Patna virus	Leaf curl disease	tomato	Plants and Fungi
649996	Tomato yellow leaf distortion virus	Leaf curl disease	tomato	Plants and Fungi
664980	Tomato leaf curl Hainan virus	Leaf curl disease	tomato	Plants and Fungi
681518	Tomato leaf deformation virus	Leaf deformation	tomato	Plants and Fungi
693894	Tomato leaf curl Cameroon virus	Leaf curl disease	tomato	Plants and Fungi
693897	Tomato yellow leaf curl Thailand betasatellite	Leaf curl disease	tomato	Plants and Fungi

743040	Tomato leaf curl Cameroon alphasatellite	Leaf curl disease	tomato	Plants and Fungi
858511	Tomato golden leaf distortion virus	Leaf curl disease	tomato	Plants and Fungi
858512	Tomato bright yellow mottle virus	Leaf curl disease	tomato	Plants and Fungi
929051	Tomato leaf curl Togo betasatellite-[Togo:2006]	Leaf curl disease	tomato	Plants and Fungi
938276	Tomato leaf curl Ranchi virus	Leaf curl disease	tomato	Plants and Fungi
938277	Tomato leaf curl Ranchi betasatellite	Leaf curl disease	tomato	Plants and Fungi
971271	Tomato leaf curl Hanoi virus	Leaf curl disease	tomato	Plants and Fungi
997078	Tomato leaf curl Comoros virus	Leaf curl disease	tomato	Plants and Fungi
1100034	Tomato leaf curl Bhatinda virus	Leaf curl disease	tomato	Plants and Fungi
1100035	Tomato leaf curl Sirsa virus	Leaf curl disease	tomato	Plants and Fungi
1129036	Tomato crinkle leaf yellows virus	Leaf curl disease	tomato	Plants and Fungi
1141722	Tomato leaf curl Togo virus	Leaf curl disease	tomato	Plants and Fungi
1161126	Tomato leaf curl New Delhi alphasatellite	Leaf curl disease	tomato	Plants and Fungi
1162280	Tomato rugose yellow leaf curl virus	Leaf curl disease	tomato	Plants and Fungi
1176156	Tomato leaf curl Yemen betasatellite	Leaf curl disease	tomato	Plants and Fungi
1231334	Tomato leaf curl Hajipur betasatellite	Leaf curl disease	tomato	Plants and Fungi
1245827	Tomato leaf curl Peru virus	Leaf curl disease	tomato	Plants and Fungi
1255765	Tomato yellow leaf curl China betasatellite	Leaf curl disease	tomato	Plants and Fungi
1260770	Tomato leaf curl Karnataka alphasatellite	Leaf curl disease	tomato	Plants and Fungi
1280308	Tomato leaf curl Guangxi betasatellite	Leaf curl disease	tomato	Plants and Fungi
1303385	Tomato leaf curl Liwa virus	Leaf curl disease	tomato	Plants and Fungi
1329381	Tomato leaf curl China virus - OX2	Leaf curl disease	tomato	Plants and Fungi
1334928	Tomato leaf curl Bhatinda betasatellite	Leaf curl disease	tomato	Plants and Fungi
1335768	Tomato yellow leaf curl Yunnan virus	Leaf curl disease	tomato	Plants and Fungi
1336597	Tomato golden leaf spot virus	Chlorotic spots	tomato	Plants and Fungi
1384631	Tomato leaf curl Gandhinagar virus	Leaf curl disease	tomato	Plants and Fungi
1384632	Tomato leaf curl Gandhinagar betasatellite	Leaf curl disease	tomato	Plants and Fungi
1391746	Tomato yellow leaf curl Saudi virus	Leaf curl disease	tomato	Plants and Fungi
1497568	Tomato leaf curl Oman betasatellite	Leaf curl disease	tomato	Plants and Fungi
1498504	Tomato leaf curl Barka virus	Leaf curl disease	tomato	Plants and Fungi
1513530	Tomato leaf curl Nigeria virus	Leaf curl disease	tomato	Plants and Fungi
1513531	Tomato yellow leaf curl Indonesia virus	Leaf curl disease	tomato	Plants and Fungi
1570110	Tomato leaf curl Gujarat betasatellite	Leaf curl disease	tomato	Plants and Fungi
1609290	Tomato leaf curl Pakistan betasatellite	Leaf curl disease	tomato	Plants and Fungi
1632184	Tomato enation leaf curl virus	Leaf curl disease	tomato	Plants and Fungi
1701549	Tomato chlorotic mottle Guyane virus	Leaf curl disease	tomato	Plants and Fungi
1761477	Tomato brown rugose fruit virus	Tomato brown rugose fruit disease	tomato	Plants and Fungi

1833825	Tomato necrotic spot associated virus	Chlorotic spots	tomato	Plants and Fungi
1955054	Tomato leaf curl Burkina Faso virus	Leaf curl disease	tomato	Plants and Fungi
2010332	Tomato leaf curl Laguna betasatellite	Leaf curl disease	tomato	Plants and Fungi
2010333	Tomato leaf curl Laos betasatellite	Leaf curl disease	tomato	Plants and Fungi
2015441	Tomato chlorotic leaf curl virus	Leaf curl disease	tomato	Plants and Fungi
2021667	Tomato leaf curl purple vein virus	Leaf curl disease	tomato	Plants and Fungi
2031200	Tomato leaf curl Nepal betasatellite	Leaf curl disease	tomato	Plants and Fungi
2033721	Tomato yellow leaf curl Yunnan betasatellite	Leaf curl disease	tomato	Plants and Fungi
2040662	Tomato yellow leaf curl Shandong betasatellite	Leaf curl disease	tomato	Plants and Fungi
2048879	Tomato leaf curl Virudhunagar alphasatellite	Leaf curl disease	tomato	Plants and Fungi
2049892	Tomato leaf curl Malaysia betasatellite	Leaf curl disease	tomato	Plants and Fungi
2169738	Tomato yellow leaf curl Shuangbai virus	Leaf curl disease	tomato	Plants and Fungi
2169845	Tomato yellow leaf curl Thailand alphasatellite	Leaf curl disease	tomato	Plants and Fungi
2203894	Tomato leaf curl Yunnan betasatellite	Leaf curl disease	tomato	Plants and Fungi
2303151	Tomato leaf curl Mahe virus	Leaf curl disease	tomato	Plants and Fungi
2560809	Tomato leaf curl Japan virus	Leaf curl disease	tomato	Plants and Fungi
2560811	Tomato leaf curl Karnataka virus 3	Leaf curl disease	tomato	Plants and Fungi
2560813	Tomato severe leaf curl Kalakada virus	Leaf curl disease	tomato	Plants and Fungi
2601257	Tomato leaf curl Mumbai virus	Leaf curl disease	tomato	Plants and Fungi
2670809	Tomato yellow leaf curl Kanchanaburi satellite	Leaf curl disease	tomato	Plants and Fungi
2740451	Tomato leaf curl Kunene virus	Leaf curl disease	tomato	Plants and Fungi
2761106	Tomato leaf curl Palampur betasatellite	Leaf curl disease	tomato	Plants and Fungi
2828343	Tomato leaf curl Chuxiong virus	Leaf curl disease	tomato	Plants and Fungi
2875796	Tomato mottle leaf distortion virus	Leaf curl disease	tomato	Plants and Fungi
2932495	Tomato leaf curl Chiayi virus	Leaf curl disease	tomato	Plants and Fungi
2932496	Tomato leaf curl Nantou virus	Leaf curl disease	tomato	Plants and Fungi
2968787	Tomato leaf curl Tianyang virus	Leaf curl disease	tomato	Plants and Fungi
2984291	Tomato leaf curl Ty Pusa virus	Leaf curl disease	tomato	Plants and Fungi
3132000	tomato yellow leaf curl Chuxiong virus	Leaf curl disease	tomato	Plants and Fungi
3145072	tomato leaf curl betasatellite Lalitpur	Leaf curl disease	tomato	Plants and Fungi
3025448	Ailanthus crinkle leaf associated emaravirus	Leaf curl disease	Toxicodendron altissimum	Plants and Fungi
598671	Trillium crinkled leaf virus	Leaf curl disease	Trillium	Plants and Fungi
32621	Tulip top breaking virus	Tulip breaking disease	Tulipa	Plants and Fungi
2761348	Tulip streak virus	Tulip streak disease	Tulipa	Plants and Fungi
421011	Veltheimia mosaic virus	Mosaic disease	Veltheimia	Plants and Fungi
1134402	Verbesina encelioides yellow vein Lakshmangarh virus	Yellow vein disease	Verbesina encelioides	Plants and Fungi
1001341	Vernonia yellow vein Fujian virus	Yellow vein disease	Vernonia	Plants and Fungi

36419	Duck influenza virus	Avian influenza	waterfowl	Vertebrates
345184	Cabbage leaf curl Jamaica virus	Leaf curl disease	wild cabbage	Plants and Fungi
217616	Grapevine leafroll virus-associated RNA6	Grapevine leafroll	wine grape	Plants and Fungi
11970	Woolly monkey sarcoma virus	Sarcoma	woolly monkeys	Primates
68416	Woolly monkey hepatitis B virus	Hepatitis A	woolly monkeys	Primates
12138	Maize chlorotic mottle virus	Leaf curl disease	Zea mays var. japonica	Plants and Fungi
1856642	Maize yellow mosaic virus	Leaf curl disease	Zea mays var. japonica	Plants and Fungi
2025388	Maize striate mosaic virus	Maize striate mosaic disease	Zea mays var. japonica	Plants and Fungi
2170101	Maize yellow dwarf virus RMV	Yellow dwarf disease	Zea mays var. japonica	Plants and Fungi

Supplementary Table 2. Consensus human virus-disease connections identified by the three LLMs.

Virus TxID	Virus name	Virus family	Disease name
10243	Cowpox virus	Poxviridae	Cowpox
10244	Monkeypox virus	Poxviridae	Monkeypox
10245	Vaccinia virus	Poxviridae	Vaccinia
10255	Variola virus	Poxviridae	Smallpox
10279	Molluscum contagiosum virus	Poxviridae	Molluscum contagiosum
10368	Human betaherpesvirus 6	Orthoherpesviridae	Encephalitis
11033	Semliki Forest virus	Togaviridae	Encephalitis
11034	Sindbis virus	Togaviridae	Sindbis fever
11037	Venezuelan equine encephalitis virus (strain TC-83)	Togaviridae	Venezuelan equine encephalitis
11039	Western equine encephalitis virus	Togaviridae	Equine encephalitis
11072	Japanese encephalitis virus	Flaviviridae	Japanese encephalitis
11087	Tick-borne encephalitis virus (STRAIN SOFJIN)	Flaviviridae	Tick-borne encephalitis
11966	Aids-associated retrovirus	Retroviridae	Acquired immunodeficiency syndrome (AIDS)
11983	Norwalk virus	Caliciviridae	Gastroenteritis
12098	Human hepatitis A virus Hu/Australia/HM175/1976	Picornaviridae	Hepatitis A
12475	Hepatitis delta virus	Kolmioviridae	Hepatitis D
33774	Hepatitis E virus (strain Pakistan)	Hepeviridae	Hepatitis E
37296	Human gammaherpesvirus 8	Orthoherpesviridae	Kaposi's sarcoma
39137	Tick-borne flavivirus	Flaviviridae	Tick-borne encephalitis
42097	Isla Vista hantavirus	Hantaviridae	Hantavirus Pulmonary Syndrome
47301	Muleshoe hantavirus	Hantaviridae	Hantavirus Pulmonary Syndrome
64316	Russian Spring-Summer encephalitis virus	Flaviviridae	Russian Spring-Summer encephalitis
77763	Banna virus	Sedoreoviridae	Febrile illness
95342	Sapporo virus	Caliciviridae	Gastroenteritis
99000	Tanapox virus	Poxviridae	Tanapox
118043	Untyped human rhinovirus OK88-8162	Picornaviridae	Common cold
178534	JC virus x BK virus	Polyomaviridae	Progressive multifocal leukoencephalopathy (PML)
205488	Ebola virus sp.	Filoviridae	Ebola virus disease
224649	Oyster norovirus	Caliciviridae	Gastroenteritis
235406	untyped human enterovirus	Picornaviridae	Meningitis
261202	Alto Paraguay hantavirus	Hantaviridae	Hantavirus Pulmonary Syndrome
315431	untyped Human adenovirus	Adenoviridae	Conjunctivitis
318275*	Untyped Human T-lymphotropic virus	Retroviridae	Adult T-cell leukemia/lymphoma
318275*	Untyped Human T-lymphotropic virus	Retroviridae	HTLV-1-associated myelopathy/tropical spastic paraparesis
374990	Mayaro virus (strain Brazil)	Togaviridae	Mayaro fever
430511	Juquitiba virus	Hantaviridae	Hantavirus Pulmonary Syndrome

467146	Cowpox-Vaccinia virus	Poxviridae	Cowpox
493803	Merkel cell polyomavirus	Polyomaviridae	Merkel cell carcinoma
650132	Aichi virus A846/88	Picornaviridae	Gastroenteritis
1095764	Caliciviridae Hu/GII.4/Xiamen/May0511/2010/CHN	Caliciviridae	Gastroenteritis
1244521	ANAJ Hantavirus	Hantaviridae	Hantavirus Pulmonary Syndrome
1244522	CASV Hantavirus	Hantaviridae	Hantavirus Pulmonary Syndrome
1244523	CASV-2 Hantavirus	Hantaviridae	Hantavirus Pulmonary Syndrome
1314973	Lianghe virus	Hantaviridae	Hemorrhagic Fever with Renal Syndrome (HFRS)
1518574	Burkina Faso astrovirus	Astroviridae	Gastroenteritis
1933189	Sandfly fever Naples phlebovirus	Phenuiviridae	Sandfly fever
1933190	SFTS phlebovirus	Phenuiviridae	Severe Fever with Thrombocytopenia Syndrome (SFTS)
2169701	Onyong-nyong virus	Togaviridae	Onyong-nyong fever
2304450	Dakrong virus	Hantaviridae	Hantavirus Pulmonary Syndrome
2558248	Adenoviridae sp.	Adenoviridae	Conjunctivitis
2748762	Colorado tick fever coltivirus	Spinareoviridae	Colorado tick fever
2778957	Biya River virus	Hantaviridae	Hemorrhagic Fever with Renal Syndrome (HFRS)
2994337	Human hepatitis E virus genotype 3	Hepeviridae	Hepatitis E
3040071	Human orthopneumovirus subgroup A	Pneumoviridae	Bronchiolitis
3228684	Sager Creek orthohantavirus	Hantaviridae	Hantavirus Pulmonary Syndrome

*One virus causing two distinct diseases are highlighted
